# Editorial: Antiviral drug discovery against pathogens of pandemic concern: Advancements in target site identification and structure-based drug development

**DOI:** 10.3389/fmolb.2023.1165208

**Published:** 2023-03-09

**Authors:** Krishna M. Padmanabha Das

**Affiliations:** ^1^ Department of Biological Chemistry and Molecular Pharmacology (BCMP), Harvard Medical School, Boston, MA, United States; ^2^ Cancer Biology, Dana-Farber Cancer Institute, Boston, MA, United States

**Keywords:** anti-viral drug discovery, structure-based drug design, pandemic viruses, SARS-CoV-2, broad spectrum antivirals, Lassa virus, polymyxovirus, pandemic preparedness

The recent emergence of multiple zoonotic viruses such as Ebola virus and severe acute respiratory syndrome coronavirus 2 (SARS-Cov-2) have demonstrated that viruses of animal origin are a realistic threat to global public health. One of the pressing questions at the moment is how prepared we are in terms of development of therapeutic strategies in case of another pandemic. Assessing the risk of animal-to-human spillover for each of the viruses and identifying the targets for countermeasure initiatives can in long run reduce the economic and health impacts of emerging diseases. While it is incontestable that viruses need to be prioritized, for a focused global drug discovery strategy, based on their pandemic potential, ranking the risk of animal-to-human spillover is not an easy task. There have been several attempts to rank viruses based on their zoonotic potential, host, various factors about the virus, environment and related human behavior (Trock et al., 2015; Geoghegan et al., 2016; Olival et al., 2017). According to a recently developed viral risk ranking framework, Lassa virus, SARS-CoV-2, Ebola virus, Seoul virus, and Nipah virus are among the viruses that have the highest risk of zoonotic jump (Grange et al., 2021). Interestingly, several of the viruses that are identified as viruses of pandemic potential are RNA viruses (Carrasco-Hernandez et al., 2017). RNA viruses are characterized by compact genomes and a high mutation rate (10^−3^–10^−5^ errors per nucleotide and replication cycle), generating changes-which can usually occur due to replication errors as well as part of the evolutionary mechanisms of viruses (Dolan et al., 2018).

While big data and machine learning have generated several methodologies that could be useful for infectious diseases intelligence, a parallel stream of efforts to identify effective druggable targets in these pathogens, and discovery and development of therapeutic agents against these targets could be crucial for an effective pandemic preparedness strategy. Drugs that can help in preventing a pandemic, should be suitable for post-exposure prophylaxis, possess oral activity with a good safety profile, have an affordable cost, and be easy to deliver and suitable for being used in combination with other drugs. The global research community consisting of the scientists from academia, industry and non-profits will have to act in a collaborative manner to generate a toolkit of antiviral agents covering a number of viral families. This Research Topic is one of such efforts to bring research efforts happening in this direction under one umbrella. The early efforts of curbing SARS-CoV-2 revolved around repurposing existing approved drugs and the results from these give us several lessons on how to rapidly develop antiviral drugs in case of a pandemic outbreak (Namchuk, 2021). Pfizer leveraged their work on SARS-CoV-1, where they had previously developed an antiviral called PF-00835231, which was originally derived from rupntirivir developed against rhinovirus protease. Even though the SARS-CoV-1 and rhinoviral proteases are not very similar, the similarities in their peptide substrates meant that Pfizer could modify rupntirivir to develop PF-00835231, which proved to be an effective covalent inhibitor. Since SARS-CoV-1 and SARS-CoV-2 Mpros are highly homologous, Pfizer could quickly advance PF-00835231 analogs to clinical trials (Vries et al., 2021) which eventually resulted in the FDA approval of paxlovid. Similarly, a broadly acting antiviral agent named molnupiravir, originally developed against Venezuelen equine encephalitis virus (VEEV) was repurposed to be used as treatment against SARS-CoV-2 (Painter et al., 2021) which was clinically developed by Merck and acquired an emergency use authorization (EUA) from FDA. Even though rapid optimization of existing antivirals could be fruitful in case of pandemic outbreak, novel specific antiviral could be much more effective. Novel specific antiviral discovery is needed across the whole spectrum of virus families that have a pandemic potential, so that when a new threat emerges, the scientific community will be in a position to evaluate the options in rapid clinical trials.

**FIGURE 1:**
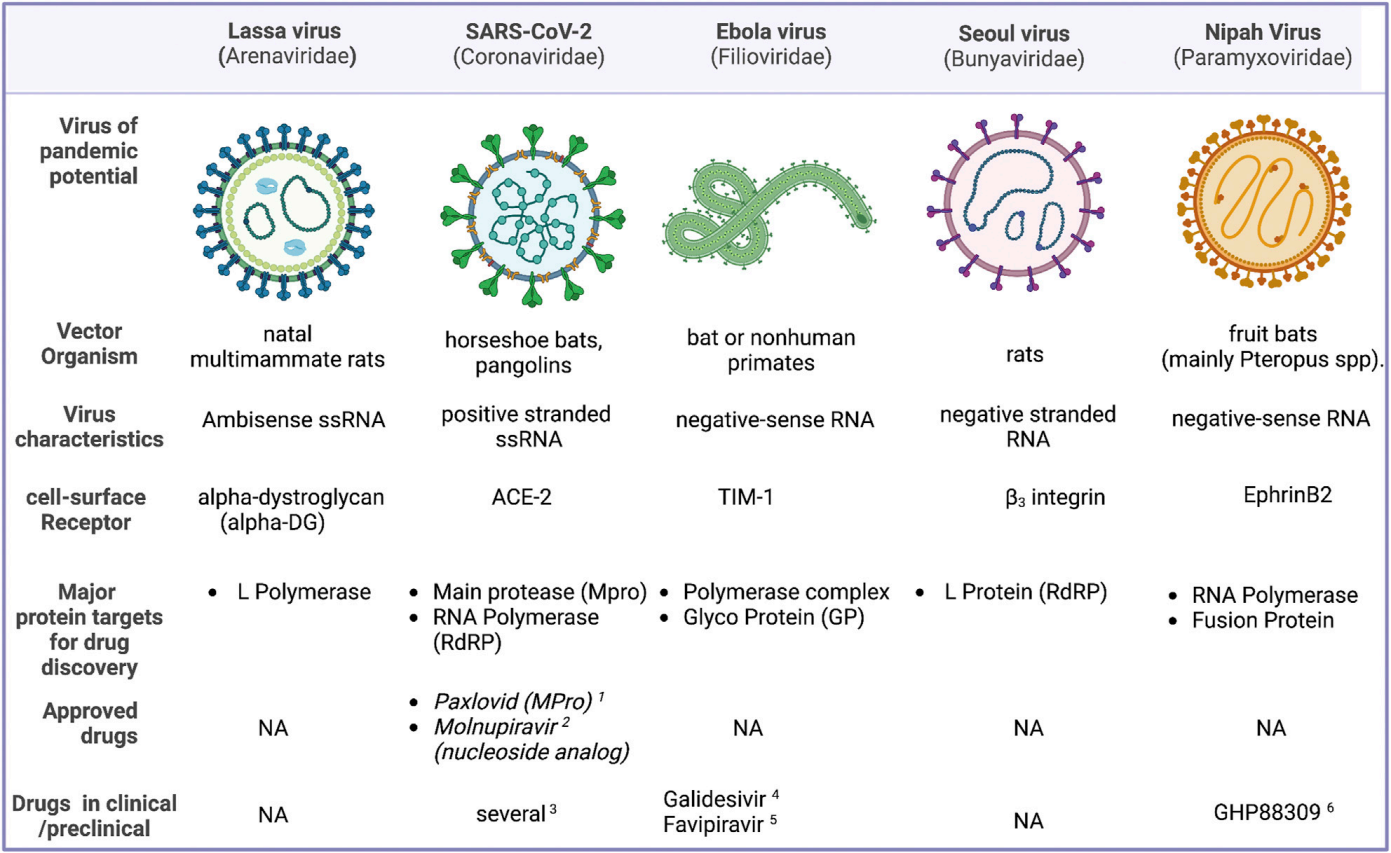
An overview of some of the viruses that are considered to have a high potential to cause a pandemic, their structural features, major targets for drug development and potential drug candidates. 1) (Owen et al., 2021). 2) (Kabinger et al., 2021). 3) (Bolarin et al., 2021). 4) (Julander et al., 2021). 5) (Bixler et al., 2018). 6) (Cox et al., 2020).

Antiviral small molecule drugs could be disrupting one of the several steps involved in the viral cycle. Currently existing antiviral agents can be 1) viral entry blockers 2) Preventing viral replication and curtailing the viral load (protease inhibitors, reverse transcriptase, and polymerase inhibitors both nucleoside analogs and non-nucleoside inhibitors, integrase inhibitors, etc.) 3) disrupting the ability of the viral pathogen to subvert host defenses 4) inhibitors of viral assembly or packaging. One another class of antivirals agents are those which target the host proteins. While some of the targets such as the proteases and RdRP are well characterized there are several targets which can be targeted in principle, but the targets are not experimentally validated. In addition to conventional active site inhibitors, critical protein-protein interfaces could also be targeted. Figure 1 provides an overview of the top viral agents that has the potential to cause a pandemic, and their structural features and status of drug development. This Research Topic is an attempt to consolidate the advancement in research towards antiviral discovery, which would prepare us to face a future viral pandemic more effectively.

RNA viruses continue to evolve mainly through mutations that enable them to bind better to the host receptor, or alter the infection rate or virus-host interaction, eventually turning out to be more contagious and at times with increased pathogenicity. Insights into the possible mutations could be very helpful in predicting future pandemics, as well as for developing effective vaccine designs. Rhoades et al. carried out a detailed investigation of missense mutations in the MERS-CoV S protein and its effects on protein stability and binding affinity with DPP4. The authors found several mutations that could affect the stability of the S protein as well as its binding affinity to the DPP4 receptor. These observations could be useful not only in case of MERS-CoV, but also for SARS-CoV and SARS-CoV-2 as the authors have identified equivalent key positions in these viruses as well. Alegría-Arcos et al. (2022) have utilized a network pharmacology workflow to investigate complex protein-protein interactions of both viral and host proteins, as well as protein-drug interactions to recommend some approved drug candidates that are worth moving forward to clinical trials. While this strategy can be an effective way to identify repurposed drugs for SARS-CoV-2, this could also be implemented quickly in case of an emerging pandemic.

One of the possible ways to inhibit SARS-CoV-2 infection is to develop inhibitors that block the entry of the virus by disrupting the protein-protein interaction between the viral spike protein and the human cellular receptor ACE-2. Odolczyk et al. (2021), in their previous work had developed shorter peptides derived from the alpha peptide of ACE-2 with nanomolar affinities. In the current work, Odolczyk et al. have further improved these short peptides, and have optimized the peptides to have an affinity one magnitude higher than the previous version. This peptide-based strategy has potentials to be developed to drugs in a fast-paced manner, and could be applied to develop antiviral peptides against other emerging viral diseases.

Long non-coding RNAs (lncRNAs), the RNA transcripts that are over 200 nucleotides are known to have varying functions ranging from regulating gene specific transcription to post-transcriptional regulation to epigenetic regulation (Mattick et al., 2023). In this interesting review, Zhong et al. have consolidated various research works on lncRNAs to assess their suitability as therapeutic targets as well as to validate their use as biomarkers for viral infections. The authors have observed that lncRNA profiles especially NEAT1 and MALAT1, are distinct between healthy, non-severely affected and severely affected patients. The increased presence of the lncRNAs in patient’s lung samples, and even easily accessible saliva and nasopharyngeal swab samples make them ideal biomarkers. Zong et al. also point out the importance of lncRNAs in hyperactivation of the immune system such as cytokine release syndrome (CRS) and suggest that targeting lncRNAs could be beneficial to develop drugs that can prevent the high mortality rate of respiratory viral diseases.

All in all, in this topic we present a bouquet of studies that range from prediction of possible spike mutations in MERS-CoV (Rhoades et al.), a novel network pharmacology workflow to identify drugs for repurposing (Alegría-Arcos et al., 2022), an effective way to rapidly develop high affinity peptide based antiviral therapy that inhibit viral entry (Odolczyk et al.) and insights on use of lncRNAs biomarkers as well as drug targets in viral diseases (Zhong et al.). These are all exciting works from the perspective of pandemic preparedness, and we hope would be of interest to virologists, structural biologists, and scientists working towards antiviral drug discovery in both industry and academia.
